# Zoonotic Tick-Borne Pathogens in Ticks from Vegetation and Alpine Ibex (*Capra ibex*) in the Maritime Alps, Italy

**DOI:** 10.3390/ani14152251

**Published:** 2024-08-02

**Authors:** Arianna Menzano, Paolo Tizzani, Marisa Diana Farber, Aitor Garcia-Vozmediano, Laura Martinelli, Luca Rossi, Laura Tomassone

**Affiliations:** 1 Ente di Gestione delle Aree Protette delle Alpi Marittime, 12010 Valdieri, Cuneo, Italy; arianna.menzano@gmail.com (A.M.); laura.martinelli@areeprotettealpimarittime.it (L.M.); 2 Department of Veterinary Sciences, University of Turin, 10095 Grugliasco, Turin, Italy; paolo.tizzani@unito.it (P.T.); aitor.garciavozmediano@izsto.it (A.G.-V.); luca.rossi@unito.it (L.R.); 3 Instituto de Agrobiotecnología y Biología Molecular (IABIMO) Inta-Conicet, Hurlingham 1686, Argentina; farber.marisa@inta.gob.ar

**Keywords:** ticks, Alpine ibex, Alps, *Anaplasma phagocytophilum*, Borrelia burgdorferi s.l., zoonoses

## Abstract

**Simple Summary:**

Ticks are spreading at high altitudes in mountain areas and can come into contact with previously unexposed or poorly exposed hosts, such as Alpine ibex (*Capra ibex*). We collected ticks on ibex and vegetation in the northwestern Italian Alps and tested them for tick-borne pathogens. *Ixodes ricinus* was the most abundant tick species collected and was infected with *Borrelia burgdorferi* s.l., *Rickettsia* spp. and *Anaplasma phagocytophilum.* Our results suggest that Alpine ibex, like other wild ungulate species, may act as a reservoir for *A. phagocytophilum* while being an incompetent reservoir for the Lyme borreliosis agent. Future studies should monitor the possible impacts of ticks and transmitted pathogens on the health and conservation of ibex.

**Abstract:**

In the Maritime Alps (northwestern Italy), we collected ticks from vegetation and Alpine ibex (*Capra ibex*). *Ixodes ricinus* was the most abundant species in the study area, questing up to 1824 m a.s.l. and infesting 28 out of 72 ibexes. *Haemaphysalis punctata, H. sulcata* and *Dermacentor marginatus* were also collected. The abundance of questing ticks significantly decreased with altitude, with beechwoods being the preferred habitat. By PCR, we identified *Borrelia burgdorferi* s.l. in questing *I. ricinus* (28.3%; 95%CI: 19.4–38.6) but not in specimens collected from animals. *Rickettsia* spp. infected both questing (20.6%; 95%CI: 12.9–30.3) and on-host (30.2%; 95%CI: 21.2–40.4) *I. ricinus. Anaplasma phagocytophilum* was detected in 4.3% (95%CI: 1.2–10.8) of questing *I. ricinus* and in 45.3% (95%CI: 34.6–56.4) of *I. ricinus* collected from ibex. Female *I. ricinus* collected on animals were significantly more infected with *A. phagocytophilum* than females collected from vegetation (OR = 11.7; 95%CI: 3.8–48.1). By amplifying and sequencing a fragment of the *groEL* gene, we identified 13 *groEL* haplotypes, clustering with ecotypes I and II; ecotype I, prevalent in our sample, is considered zoonotic. Our study demonstrates the presence of different tick-borne zoonotic agents in the study area, encompassing a wide altitudinal range, as confirmed by the ticks found on ibex, a typical mountain-dwelling mammal. The results also confirm the altitudinal range expansion of ticks and associated pathogens in the Alps and suggest that Alpine ibex may act as a reservoir for *A. phagocytophilum*, as do other wild ungulate species.

## 1. Introduction

Tick-borne diseases have been increasingly reported in the western Italian Alps in recent years. This increase may be attributed to climatic and environmental changes, resulting in a greater abundance of vectors, particularly *Ixodes ricinus*. This much investigated tick is now found at unusual altitudes and in districts where it was previously rare or not reported [1].

Wild ungulates are key in the maintenance and spread of tick populations and can also act as amplifiers or reservoirs for tick-borne pathogens [2]. However, the extent of tick infestation in ungulate hosts is modulated by factors such as habitat preferences, feeding and resting behavior and the use of space in general [3]. 

Alpine ibex (*Capra ibex*), a mountain-dwelling caprine (subfamily Caprinae), typically resides above the tree line for most of the year. With rare exceptions, it is only in spring that ibexes descend to lower altitudes to take advantage of the earlier vegetation regrowth. In this season, it is not rare to observe ibexes browsing in shrubs and sparse forest patches at short distances from the particularly attractive valley meadows [4]. Despite this, ibexes are considered at lower risk of tick exposure than wild ruminants with a year-long preference for forest habitats such as roe deer, red deer and, to a lesser extent, northern chamois [5]. Records of tick infestation in Alpine ibex are infrequent. Interestingly, a survey conducted in the 1980s in the Maritime Alps, northwestern Italy, reported the presence of three tick species, *Ixodes ricinus, Haemaphysalis punctata* and *Dermacentor marginatus*, on live and dead Alpine ibex [6].

As part of the ALCOTRA LEMED-IBEX project (2017–2020) focused on monitoring, management and conservation of ibex in the Alps (https://www.interreg-alcotra.eu/it/lemed-ibex accessed on 26 July 2024), we investigated the occurrence of ticks and associated tick-borne pathogens (TBPs) on ibex and vegetation in the Maritime Alps. Our study aimed at assessing the distribution, abundance and diversity of ticks and zoonotic TBPs in the area, as well as the environmental risk factors associated with tick presence. Finally, we aimed to evaluate the potential role of ibex in the transmission cycle of tick-borne bacteria, given the increasing tick abundance in the Alps linked to ongoing climate and environmental changes. 

## 2. Materials and Methods

### 2.1. Study Area 

The IT1160056 Alpi Marittime Special Area of Conservation (Nature 2000 network) is located at the southwestern end of the Alpine chain, between two important international passes: Colle di Tenda (1871 m a.s.l.—44°8′57″ N 7°33′43″ E) and Colle della Maddalena (1996 m a.s.l.—44°25′18″ N 6°53′55″ E). It covers an area of over 33,000 hectares and includes three valleys in the Piedmont region (Gesso, Vermenagna and Stura). The territory is characterized by 24 peaks exceeding 3000 m a.s.l., around 80 lakes of glacial origin and 7 small glaciers, the southernmost in the Alps. 

During winter months, snow accumulation varies from 300 to 400 cm per year at around 1000 m a.s.l and up to 700–800 cm per year above 2000 m a.s.l. [7]. The annual average cumulative snow has shown a stationary trend, while the resident time of snow has progressively reduced by approximately 20 days from 1980–2000 to 2000–2018. At present, snow cover persists for approximately 170/200 days at altitudes above 2000 m a.s.l., around 130 days for areas between 1500 and 2000 m a.s.l. and nearly 50 days in areas below 1500 m a.s.l. The reduction in snow residence time and the resulting decrease in ice coverage are attributed to the increase in global temperatures [8]. In the Maritime Alps, an increase in the annual maximum temperatures was observed at altitudes above 700 m a.s.l.; in particular, an increase of 0.73 °C every 10 years was registered over the period 1981–2018 [7]. Precipitations shows slight variation over the years, ranging from 960 mm per year in the lowland territories to over 1,500 mm per year recorded at the Limone Pancani station (Vermenagna Valley); such strong variation is due to the proximity of the Mediterranean Sea, which is only 50 km from the highest peaks of the Maritime Alps [9]. 

The geographical position, morphology and geological events have made the Maritime Alps a biodiversity hotspot and a habitat suitable for all six Alpine ungulate species: northern chamois (*Rupicapra rupicapra*), roe deer (*Capreolus capreolus*), wild boar (*Sus scrofa*), red deer (*Cervus elaphus*), mouflon (*Ovis aries musimon*) and Alpine ibex.

### 2.2. Tick Collection 

Ticks were collected from vegetation and ibexes. Questing ticks were collected in 30 transects of 100 m length, which were randomly selected using Qgis 3.4.3 [10], considering the altitudinal range (from 780 to 1824 m a.s.l.) and three habitat types: beechwood (*Fagus sylvatica*), sprucewood (*Picea abies*) and stone-pinewood (*Pinus cembra*). Transects were covered by an operator equipped with a dragging cloth (size: 1 × 1 m), with stops every 25 m. At each stop, the nymphs and adults present on the dragging cloth were counted and stored in vials containing 70% alcohol; larvae were not collected. The transects were walked once a month from September to October 2018 and from May to October 2019.

Animals were captured within a capture–mark program to assess the dynamics and health status of ibex populations in the western Alps in the framework of the ALCOTRA LEMED-IBEX project [11]. Captures were carried out in June and July 2018 (*n* = 61) and in May and June 2019 (*n* = 11) at an altitude ranging between 1515 and 2848 m a.s.l. The animals were anesthetized for manipulation and visually inspected for ticks during a limited time frame (approximately 5 min per animal). Feeding ticks were extracted using tweezers and stored in labeled vials containing 70% alcohol until identification.

### 2.3. Laboratory Analyses 

Collected ticks were identified morphologically by using a stereomicroscope and identification keys [12,13]. DNA was extracted from individual ticks by using the DNAzol reagent^®^ (Life Technologies Ltd., Warrington, UK). To compare tick-borne bacterial infection between questing and on-the-host *I. ricinus*, we tested female specimens recovered from both vegetation and animals. A total of 68 females collected by dragging were tested, with 1 being excluded due to poor conservation. Additionally, a sample of 87 females collected from ibex was tested, selecting 1 to 15 ticks from each animal based on the availability. Additional specimens of *I. ricinus* (males and nymphs) from the environment and animals were tested. 

We investigated the presence of tick-borne pathogens using conventional PCR targeting the intergenic spacer region 5S and 23S rRNA of *Borrelia burgdorferi* s.l. [14] as well as a fragment of the rickettsial genes *gltA* [15] and *OmpA* [16]. *Anaplasma phagocytophilum* was identified using a qPCR assay to target the *msp2* gene [17] followed by an endpoint *groEL* gene nested PCR [18]. Positive and negative water controls were used in all molecular assays. 

The ExoSAP-IT™ PCR Product Clean-up Kit (GE Healthcare Limited, Chalfont, UK) was used to purify the PCR amplicons, which were then sent to an external service (BMR Genomics, Padua, Italy) for Sanger sequencing, using the same PCR primers.

Nucleotide sequences were handled by using BioEdit [19] and the resulting FASTA file was used for further sequence analysis. The sequences obtained were compared with sequences available in the National Center for Biotechnology GenBank^®^ database (nucleotide collection, nr/nt) with the BLASTn tool [20]. 

The genetic analysis of the *groEL* gene fragment from *A. phagocytophilum* was performed according to Jahfari et al.’s ecotype classification [17] following the haplotype definition implemented by Jaarsma and co-authors [21]. Using MUSCLE [22], we aligned our *groEL* sequences to 280 sequences representative of *A. phagocytophilum* ecotypes originating from *I. ricinus* and wild ruminants from countries in the Alpine region (Supplementary Table S1 in [21]). In detail, we sorted the database by country of collection (Italy, France, Switzerland, Austria, Germany, Slovenia) and by source of isolate (ibex, red deer, roe deer, mouflon, chamois). We then constructed a statistical TCS haplotype network (statistical parsimony) using PopArt [23,24] to portray evolutionary relationships among the samples.

### 2.4. Statistical Analyses 

The data were analyzed using R software version 4.3.2 for Windows [25]. We carried out univariate and multivariate (generalized linear model—GLM) analyses using a negative binomial distribution to evaluate the influence of different environmental and temporal factors on the abundance of *I. ricinus* ticks in the environment: habitat type (beech forest, spruce forest, stone pine forest); slope exposure (north, south, east, west); altitude range, subdivided into three belts (a: <1000 m a.s.l.; b: 1000–1500 m a.s.l.; c: >1500 m a.s.l.); proximity to tourist sites (0 = far from tourist sites; 1 = close to tourist sites); and month of sampling. A correlation analysis to assess the distribution of ticks along an elevation range was performed using the Spearman test.

The prevalence and 95% exact binomial confidence intervals (CIs) of tick infestation in Alpine ibex were calculated using a binomial exact test. To evaluate significant differences in tick infection by TBPs in female *I. ricinus* from hosts and vegetation while considering repeated measures (ticks collected from the same hosts/in the same location), we used generalized estimating equations and calculated the odds ratio (OR) with a 95% CI. For all statistical tests, the statistical significance level was set at 0.05%.

## 3. Results

**Figure 1:**
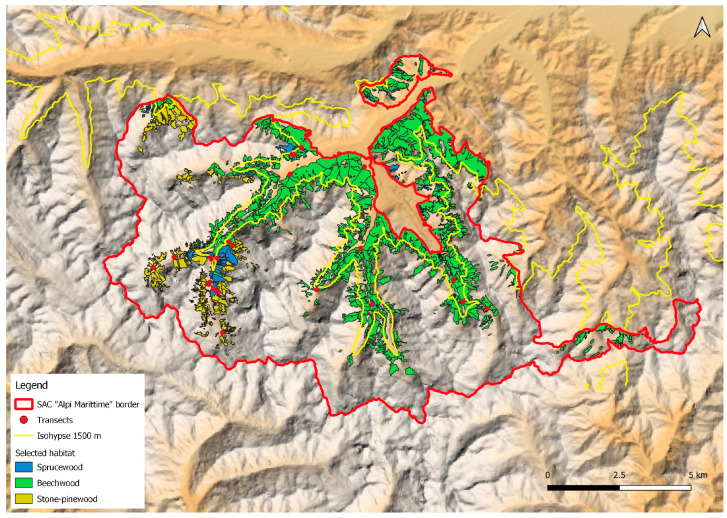
The distribution of transects for tick collection (red spots) with the dragging technique within the Alpi Marittime Special Area of Conservation in the three different habitats (sprucewood, beechwood, stone-pinewood).

A total of 874 tick specimens were collected across the 30 dragging transects (Figure 1), for a total of 180 dragging sessions. *Ixodes ricinus* was the most widespread and abundant species, accounting for 93.7% (95%CI: 91.9–95.2) of the collected ticks, including 645 nymphs, 69 females and 94 males. *Haemaphysalis punctata* and *Dermacentor marginatus* were also collected, although less frequently. Specimens of *H. punctata* accounted for 5.4% (95% CI: 4.0–7.1) of our tick sample, consisting of 33 nymphs, nine females and five males. The prevalence of *D. marginatus* was 0.7% (95%CI: 0.3–1.5), with only two nymphs, three females and one male found. Ticks were present up to an altitude of 1824 m a.s.l.

Table 1 shows the mean numbers of questing *I. ricinus* ticks per 100 m of dragging by development stage (nymphs, adults), habitat type and altitudinal range. The GLM highlighted a significant association of tick abundance with altitudinal range (with a decreasing abundance at higher altitudes) and habitat type. In particular, the tick numbers were significantly lower above 1500 m compared to the baseline altitude (<1000 m; OR: 0.31, 95%CI: 0.14–0.67) and in stone-pinewoods compared to beechwoods (OR: 0.21, 95%CI: 0.08–0.54). Details on the model results are displayed in Table S1 and Figure S1.

Ticks infested 28 out of the 72 captured ibexes (38.9%). Infested animals were captured up to 2465 m a.s.l. and 143 ticks were collected from the animals. *Ixodes ricinus* was the most abundant species, with 89 females and 36 males (87.4% of collected ticks, 95%CI: 80.8–92.4), followed by *Haemaphysalis punctata* (12 females, 4 males; 11.2%, 95%CI: 6.5–17.5). Two specimens of *Haemaphysalis sulcata* (one female, one male; 1.4%, 95%CI: 0.2–5.0) were also collected on an ibex captured in early June 2018. No *Dermacentor* spp. were collected on live-captured ibexes. A negative correlation was found between the prevalence of infested ibexes and capture altitude (rho = −0.99; *p*-value < 0.001). Ticks infested all of the ibexes captured between 1500 and 1700 m a.s.l.

As illustrated in Table 2, *Borrelia burgdorferi* s.l. was identified in 27.2% (95%CI: 18.4–37.4) of questing *I. ricinus* (25 out of 92 examined specimens). We identified *B. afzelii* (*n* = 8), *B. garinii* (*n* = 6) and *B. lusitaniae* (*n* = 6); we submitted representative sequences to GenBank^®^ (accession numbers: OR778932–4). The genospecies of five positive samples could not be determined due to low sequence quality. *Borrelia burgdorferi* s.l. was not detected in *I. ricinus* collected from animals. 

*Rickettsia* spp. was detected in both questing (20.6%; 95%CI: 12.9–30.3; *n* = 19) and on-host *I. ricinus* (23.2%; 95%CI: 16.1–31.6; *n* = 29). *Rickettsia helvetica* was the prevalent species, accounting for 77.1% of the positive samples (*n* = 37); *R. monacensis* was identified in the remaining 11 positive samples (GenBank^®^ accession no. of representative sequences: OR777613-6).

**Figure 2:**
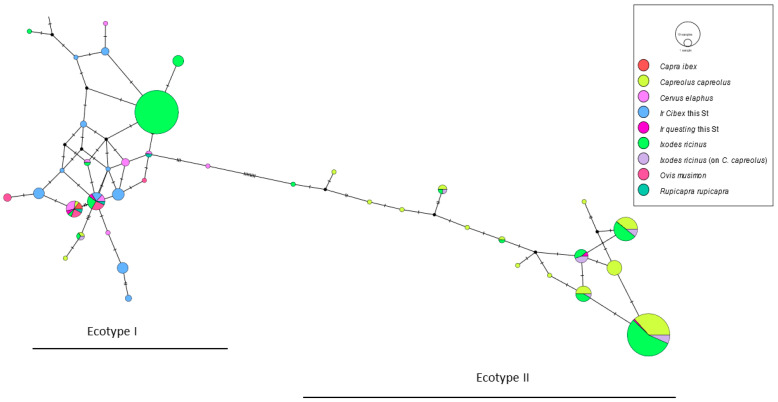
The haplotype TCS network based on *A. phagocytopilum groEL* gene fragment sequences from Alpine countries’ wild ungulates and *I. ricinus* samples. The network topology shows two distinct genetic clusters, corresponding to Ecotype I (left) and Ecotype II (right). The haplotype frequency is displayed by the circle sizes; as an example, the size of a one-sample and of a 10-samples circle is shown in the legend. The haplotypes detected in this study are indicated as ‘Ir C.ibex this St’ (*I. ricinus* feeding on ibexes, light blue) and ‘Ir questing this St’ (questing *I. ricinus*, fuchsia).

*Anaplasma phagocytophilum* was detected in 4.3% (95%CI: 1.2–10.8; *n* = 4) of questing *I. ricinus* and in 31.2% (95%CI: 23.2–40.1; *n* = 39) of *I. ricinus* collected from ibex. *Ixodes ricinus* females collected from animals were significantly more infected by *A. phagocytophilum* than females collected from vegetation (OR = 11.8; 95%CI: 3.5–39.7). From the 36 analyzed *A. phagocytophilum* sequences (4 detected in questing *I. ricinus* females, 31 in on-host females and 1 in an on-host male), we identified 13 *groEL* unique haplotypes. Out of them, two haplotypes were detected only in questing ticks; some animals hosted ticks infected by different haplotypes (Table S2). The TCS network analysis demonstrated that ecotype I was dominant, including *groEL* haplotypes from the 32 *I. ricinus* feeding on *C. ibex* and 2 questing ticks clustering together with haplotypes previously identified in several ungulate species in the Alps, including *C. ibex* haplotypes (GenBank^®^ accession no.: PP359697-729 and PP359732). On the other hand, two questing ticks grouped with haplotypes in ecotype II (GenBank^®^ accession no.: PP359730-1) (Figure 2). 

## 4. Discussion

Changing climate, host movements, increasing reforestation and the abundance of host populations are factors that may explain the increase in the altitudinal and latitudinal range of ticks in Europe, such as *I. ricinus* [26,27] and *Dermacentor reticulatus* [28]. Moreover, new tick species have been introduced, like *Hyalomma* spp. in northern European countries [29]. 

In the western Italian Alps, the spread of *I. ricinus* and *D. marginatus* at altitudes above 1000 m has been recently reported [1,30,31] together with the introduction of *D. reticulatus* [31]. Our study provides further evidence of the altitudinal rise of ticks in the Alps up to 1800 m and near the tree line. We confirmed the presence of *I. ricinus, H. punctata* and *D. marginatus* in the study area, where they had already been collected on ibex and northern chamois [6]. Unfortunately, the past study did not report tick numbers, so we are not able to compare the results and assess changes in the ticks’ abundance. We also collected a new species: *H. sulcata.* This finding suggests the suitability of the Maritime Alps’ mountain steppes for this tick. The colder climate of the study area compared to the Mediterranean areas where *H. sulcata* is normally found in Italy [14] might explain its finding in June, which is out of the normal activity peak of adults (early spring and autumn) [12,13].

We hypothesize that individual ibexes harboring ticks when sampled well above the tree line were parasitized during short-term displacements at lower altitudes where they may be observed feeding and resting in bushes and open forest habitats. In the study area, these tick-friendly habitats likely represent a favorite site for the cross-transmission of TBPs between ibex and summering livestock [32]. Even if the literature on ticks and TBPs associated with Alpine ibex is scarce, studies in the Alpine region revealed that tick-borne pathogens known to infect domestic ruminants are also found in ibex tissues: *Babesia* spp. was detected in ibex blood in Switzerland [33] and *A. phagocytophilum* was detected in ibex spleens [34] and blood [5] in Austria. 

In our study, ibex tissues were unfortunately not available to investigate TBP infection. However, we detected *A. phagocytophilum*, the agent of granulocytic anaplasmosis, in *I. ricinus* ticks feeding on the animals. The infection prevalence was significantly higher in feeding ticks compared to questing ticks, suggesting that ibexes could have a role in the endemic cycle of *A. phagocytophilum*. Other wild ungulate species, deer in particular, are considered reservoirs of this pathogen [35,36]. We could group *A. phagocytophilum groEL* sequences into 13 haplotypes, which belong to two ecotypes: I and II. All *groEL* sequences from ticks feeding on ibex belong to ecotype I (Table S2), which has been reported in several animal species and ticks [17], including ibex tissues [34]. Two ibexes hosted feeding ticks infected from two different haplotypes. Considering that ticks could be the tester of the ibex’s infection haplotype, we could hypothesize that some of the ibexes can be coinfected by multiple *A. phagocytophilum* strains. However, strains detected in feeding ticks could also originate from meals on previous hosts. Ecotype II, represented by the haplotypes 9 and 13 (Table S2), was found in two questing ticks only. 

Our results also suggest that Alpine ibex may be an incompetent reservoir for the Lyme borreliosis agent, *B. burgdorferi* s.l., being resistant to maintaining the infection and failing to infect feeding ticks, as is the case with other wild ungulate species [37,38]. In fact, we detected *B. burgdorferi* s.l. in questing *I. ricinus* (with an infection prevalence of around 30% in female ticks), while all *I. ricinus* feeding on ibex were negative. It can thus be hypothesized that ibex serum can also have borreliacidal activity like deer and other ruminant serum [39,40]. It would thus be interesting to evaluate in the laboratory the ability of the *Borrelia* genospecies found in our study area (*B. afzelii, B. garinii* and *B. lusitaniae*) to survive in ibex serum.

## 5. Conclusions

Our study provides new insights into the role of Alpine ibex in the biological cycles of tick-borne pathogens. The spread of ticks at high altitudes and their novel interactions with a previously unexposed or poorly exposed ruminant host deserve future studies to monitor the possible impacts on the health and conservation of this precious localized caprine and the eventual implications for public health.

In the Maritime Alps, environmental conditions are clearly favorable to the presence of different tick species, even at high altitudes. *Ixodes ricinus* infected by *B. burgdorferi* s.l. might represent a threat for both the personnel and the many tourists visiting the protected area. Accordingly, health professionals operating in the Maritime Alps should be aware of the hazard, and resources should be invested to inform people about methods to prevent tick bites, how to check oneself and pets after visiting high-risk areas and proper tick removal techniques. 

## Figures and Tables

**Table 1:** Mean number of *I. ricinus* nymphs and adults collected by dragging over 100m transects, by habitat type and altitude. Alpi Marittime Special Area of Conservation, 2018–1019.

		Habitat Type			Altitudinal Range (m a.s.l.)		
		Beechwood	Sprucewood	Stone-Pinewood	a (<1000)	b (1000–1500)	c (>1500)
***I. ricinus*** mean no. (min–max)	Nymphs	4.2 (0–31)	2.3 (0–13)	0.3 (0–3)	5.5 (0–31)	4.7 (0–26)	1.0 (0–9)
	Adults	0.9 (0–7)	1 (0–19)	0.08 (0–3)	1.3 (0–17)	1.6 (0–19)	0.3 (0–7)

**Table 2:** Prevalence of tick-borne pathogens identified in questing *I. ricinus* and on *I. ricinus* feeding on Alpine ibex; Alpi Marittime Special Area of Conservation, 2018–2019.

	Prevalence of Tick-Borne Pathogens (%, 95% CI)			
Tick-borne pathogens	Questing *Ixodes ricinus* (*n*)		*I. ricinus* on *Capra ibex* (*n*)	
	Nymphs (*n* = 24)	Females (*n* = 68)	Males (*n* = 38)	Females (*n* = 87)
*Borrelia burgdorferi* s.l.	20.8 (7.1–42.1)	29.4 (19.0–41.7)	0 (0–9.3)	0 (0–4.1)
	27.2 (18.4–37.4)		0 (0–2.9)	
*Rickettsia* spp.	16.7 (4.7–37.4)	22.1 (12.9–33.8)	13.1 (4.4–28.1)	27.6 (18.5–38.2)
	20.6 (12.9–30.4)		23.2 (16.1–31.6)	
*Anaplasma phagocytophilum*	0 (0–14.2)	5.9 (1.6–14.4)	5.2 (0.6–17.7)	42.5 (32.0–53.6)
	4.3% (1.2–10.8)		31.2% (23.2–40.1)	

## Data Availability

The original contributions presented in this study are included in the article/Supplementary Materials; further inquiries can be directed to the corresponding author.

## Supplementary Materials

The following supporting information can be downloaded at https://www.mdpi.com/article/10.3390/ani14152251/s1. Table S1: Results of the GLM evaluating the influence of risk factors on the abundance of *Ixodes ricinus* ticks in the environment. Figure S1: Expected abundance of *Ixodes ricinus* based on combined effect of altitudinal range and habitat type. Table S2: *Anaplasma phagocytophilum groEl* haplotypes detected in *Ixodes ricinus* ticks collected from the environment and *Capra ibex* in the Alpi Marittime Special Area of Conservation, 2018–2019.
