# Supplementary material for: Zoonotic Tick-Borne Pathogens in Ticks from Vegetation and Alpine Ibex (Capra ibex) in the Maritime Alps, Italy
Source: Animals (Basel). 2024 Aug 2;14(15):2251. doi: 10.3390/ani14152251 (PMC11311104; doi:10.3390/ani14152251)
Supplement: Supplementary file 1 [file animals-14-02251-s001.zip › Figure S1.pdf]

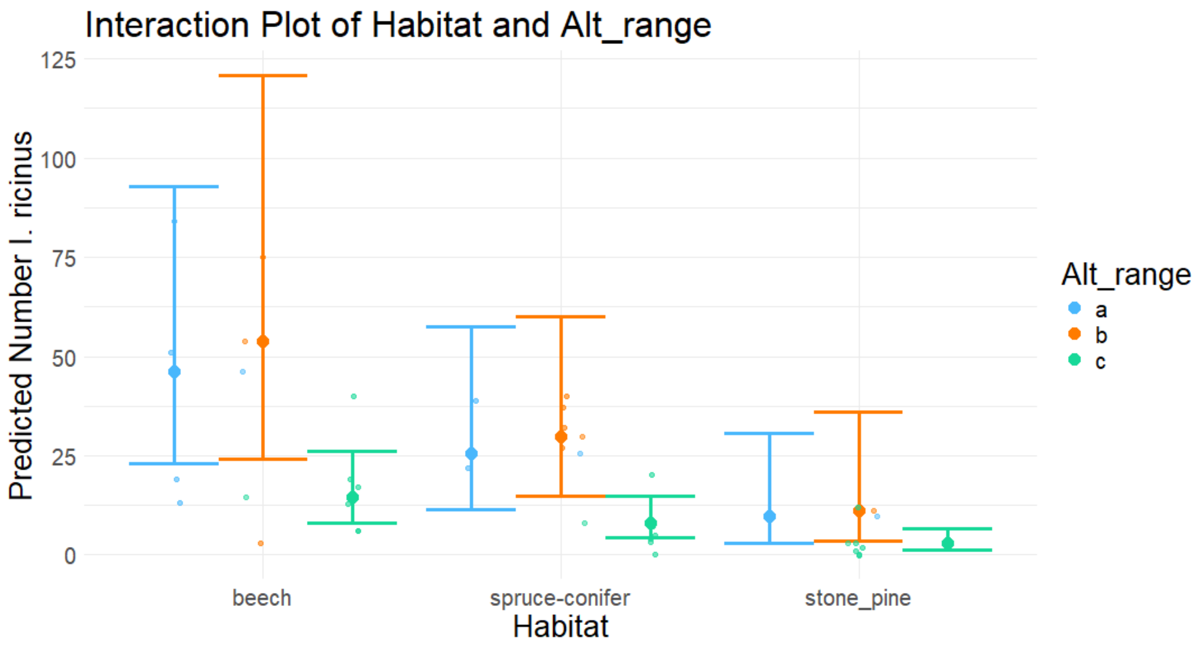

Figure S1. Expected abundance of *Ixodes ricinus* based on the combined effect of altitudinal range (a: <1000 m; b: 100-1500 m; c: >1500 m a.s.l.) and habitat type (wood type), along with 95% confidence intervals; Alpi Marittime Special Area of Conservation, 2018-2019.
