# Supplementary material for: Zoonotic Tick-Borne Pathogens in Ticks from Vegetation and Alpine Ibex (Capra ibex) in the Maritime Alps, Italy
Source: Animals (Basel). 2024 Aug 2;14(15):2251. doi: 10.3390/ani14152251 (PMC11311104; doi:10.3390/ani14152251)
Supplement: Supplementary file 1 [file animals-14-02251-s001.zip › Table S1.pdf]

term	estimate	Odds ratio (95% CI)	Interpretation	statistic (z value)
(Intercept)	3.832			11.27
Habitat spruce-conifer	-0.5949	0.55 (0.28 – 1.09)	Decrease in odds by 44.8% compared to the reference habitat (beechwood)	-1.75
Habitat stone pine	-1.569	0.21 (0.08 – 0.54)	Decrease in odds by 79.2% compared to the reference habitat (beechwood)	-3.21
Altitudinal range b	0.155	1.17 (0.46 – 3.0)	Increase in odds by 16.8% compared to the reference altitude range (altitudinal range a)	0.33
Altitudinal range c	-1.164	0.31 (0.14 – 0.67)	Decrease in odds by 68.8% compared to the reference altitude range (altitudinal range a)	-2.88

Table S1. Results of the GLM model with estimates and level of statistical significance for the factors retained in the model (Model Null deviance = 69.5; Residual deviance= 35.4; AIC= 229.3); altitudinal range a: <1000 m; b: 100-1500 m; c: >1500 m a.s.l.; Alpi Marittime Special Area of Conservation, 2018-2019.
