# Supplementary material for: Zoonotic Tick-Borne Pathogens in Ticks from Vegetation and Alpine Ibex (Capra ibex) in the Maritime Alps, Italy
Source: Animals (Basel). 2024 Aug 2;14(15):2251. doi: 10.3390/ani14152251 (PMC11311104; doi:10.3390/ani14152251)
Supplement: Supplementary file 1 [file animals-14-02251-s001.zip › Table S2.pdf]

A.phagocytophylum groEL haplotype	n of infected ticks	source of infected tick(s)		Source of coincident haplotypes*
		Environment (n of ticks)	*Capra ibex* (n of animals)	
1 (Ecotype I)	4	1	3	*R. rupricapra*, *C. elaphus*, *O. musimon*, *I. ricinus* (Nested haplotype 35*)
2 (Ecotype I)	7		2	
3 (Ecotype I)	2		1	
4 (Ecotype I)	3		2	
5 (Ecotype I)	6		2	
6 (Ecotype I)	6		1	
7 (Ecotype I)	2		1	
8 (Ecotype I)	1	1		*C. ibex*; *C elaphus*; *O. musimon*; *R. rupicapra*; *C capreolus*; *I. ricinus* (Nested haplotype 36*)
9 (Ecotype II)	1	1		*C. capreoulus*; *I.ricinus* (Nested haplotype 115*)
10 (Ecotype I)	1		1	
11 (Ecotype I)	1		1	
12 (Ecotype I)	1		1	
13 (Ecotype II)	1	1		
Total	36	4	15	

Table S2. *Anaplasma phagocytophilum groEl* haplotypes detected in *I.ricinus* ticks collected from the environment and *Capra ibex in Alpi Marittime Special Area of Conservation, 2018-2019*. * These haplotypes were previously reported by Jaarsma et al. [20]
